# Water in Mesoporous Confinement: Glass-To-Liquid Transition or Freezing of Molecular Reorientation Dynamics?

**DOI:** 10.3390/molecules24193563

**Published:** 2019-10-01

**Authors:** Wilfried Schranz, Viktor Soprunyuk

**Affiliations:** 1 Faculty of Physics, University of Vienna, Boltzmanngasse 5, 1090 Wien, Austria; viktor.soprunyuk@oeaw.ac.at; 2 Erich Schmid Institute of Materials Science, Austrian Academy of Sciences, Jahnstraße 12, 8700 Leoben, Austria

**Keywords:** mesoporous silica, supercooled confined water, glass transition

## Abstract

The first mechanical relaxation measurements (f = 400 Hz) of water confined in micro-porous silica were performed more than 40 years ago. The authors reported a so called “capillary transition” (here denoted as P3) of water in the core of the pores and a second one at a lower temperature, which they called the “adsorbate transition” (P1 in present work) related to water near the surface of the pores. The capillary transition was identified with the freezing of water in the centre of the pores. However, even 40 years later, the origin of the adsorbate transition is not yet clear. One study relates it to the liquid-to-glass transition of the supercooled water in the pores, and another study to the freezing of the proton reorientations at the lattice defects. The present work shows the data from extensive dynamic mechanical analysis (DMA) measurements (f = 0.1 Hz–70 Hz) of water confined in mesoporous silica (d = 2.5, 5 and 10 nm), which are in favour of a liquid-to-glass scenario.

## 1. Introduction

The behaviour of water in porous confinement is not only interesting from an academic point of view, but is also of substantial importance in the context of materials technology, e.g., concerning the frost damage in buildings, natural environments, the cryogenic cooling of bio-materials, etc. Despite its simple molecular structure, water offers a surprising amount of complexity [1]. Depending on the pressure and temperature, it can exist in many different long-range, ordered structures (Ices) [2] and even the liquid state, water is far from being simple [3,4].

For example, there is a question if a high-density liquid (HDL) and a low-density liquid (LDL) coexist [5,6,7,8] in a certain range of pressure and temperature above a so-called liquid-liquid critical point (LLCP) at approximately ≈ 180 K at 2.1 kbar. Another question concerns the search for possible glass transitions in water. More than 30 years ago, Mishima, et al. [9] found two distinct forms of amorphous ices, i.e., a low-density glass (ρ = 0.94 g/cm^3^), which is similar (in local structure) to crystalline ice, and a high-density glass (ρ = 1.15 g/cm^3^). Closely connected to this observation is the possibility of liquid-to-glass transitions between the corresponding phases, i.e., HDL (high density liquid)—HDA (high density amorphous) and LDL (low density liquid)—LDA (low density amorphous). The first evidence for a liquid–glass transition in LDA dates back to the calorimetric work of Johari, et al. [10] on hyperquenched water. Since that time, the bulk glass transition temperature of water has been set at T_g_ ≈ 136 K. However, later serious doubts [11,12] on the validity of water´s T_g_ ≈ 136 K were raised. The authors of [11] suggested that the small anomaly observed at approximately T_g_≈ 138 K [10] by differential scanning calorimetry (DSC) of amorphous solid water (ASW) is not the T_g_ endotherm but a shadow-T_g_ peak appearing in the tail of the real, but due to the crystallization—non-realized glass transition appearing at a higher temperature. In fact, Angell proposed that T_g_ of bulk water is between 165 K and 180 K.

Overtime, some studies started to suggest higher and higher glass transition temperatures. The highest one was proposed by Oguni, et al. [13], who set T_g_ to 210 K. As is shown below, such a high T_g_ is inconsistent with many published data [14], including ours.

The dispute on the glass transition of water still continues [15]. Some authors [16] propagated the view that the glass transition of water is in fact an (unrealized due to crystallization) order-disorder transition, that occurs in the range between 150 K and 250 K. In this picture, the calorimetric anomaly, which by most studies interpreted as the glass transition at 136 K, is then related to freezing of the remaining orientation degrees of freedom, similar to the case [17] in some molecular crystals, like e.g., C_60_.

A similar reorientation scenario contrary to a glass-to-liquid transition in water was also recently proposed, based on calorimetric and X-ray powder diffraction measurements of LDA and HDA compared with hydrogen-disordered ice VI. Another study came to the conclusion [18] that the calorimetric anomalies in the amorphous ices, which have been interpreted as liquid-to-glass transitions, are in fact governed by the thermal freezing of re-orientational motions of water molecules and at that temperature, are already located at fixed positions. The freezing of translational diffusion would then occur at a higher temperature, which that study vaguely identified with the strong-to-fragile transition (SFT) [19,20] observed in bulk water at approximately T**_FS_** ≈225 K, where the relaxation time changes from Arrhenius to Vogel-Fulcher behaviour.

Unfortunately, an experimental test to distinguish between these mentioned scenarios is strongly hampered by the fact that bulk water inevitably crystallizes [21] into hexagonal ice below 235 K by homogeneous nucleation. However, in most cases, water already freezes at higher temperatures by heterogeneous nucleation due to impurities.

One possibility to suppress crystallization completely, is to confine water in pores smaller than a critical size of d* ≈ 1 nm [22], which reflects the disability of water molecules to form tetrahedral ice structures in such small pores. For larger pore sizes there is broad consensus [23] that with cooling a fraction of water transforms into ice (i.e., the so-called hybrid ice with stacks of cubic and hexagonal layers), while the other part remains liquid down to very low temperatures [24,25,26]. Upon heating, the ice core melts, showing a pore size (R=pore radius) dependent melting temperature that follows a modified Gibbs-Thomson equation T_m_(R) = T_m_^bulk^ – K_GT_/(R − h), where T_m_^bulk^ = 273.15 K, K_GT_ = 53 ± 1 K nm and h≈ 0.4–0.7 nm. In this relation h, has been interpreted [27] to be the thickness of an unfrozen liquid layer between the confined ice and the pore wall. Recently, these parameters K**_GT_** and h were remeasured by nuclear magnetic resonance (NMR) cryoporometry [28], yielding K**_GT_**= 49.53 K nm and h = 0.533 ± 0.062 nm, which corresponds to approximately two layers of unfreezable water molecules.

Despite the large amount of knowledge about e.g., the pore size dependence of freezing and melting of water, less consistency was achieved for the dynamical behaviour of water and ice under confinement [29,30]. The present study shows how mechanical relaxation measurements can contribute to a possible solution of the problem.

This study is the third one in a sequence of dynamic mechanical analysis (DMA) investigations of water confined in small pores. In foregoing works [31,32], this study measured the dynamic mechanical response of water confined in Vycor and Gelsil with pore diameters d=10 nm (V10), 5 nm (G5) and 2.5 nm (G2), respectively. On samples fully filled with water, three anomalies were identified in the data at three different temperatures, which was related to three processes, denoted as P1, P2 and P3. The anomaly P3—which shows all characteristics of a first order phase transition (hysteresis between freezing T**_f_** and melting T**_m_**, no frequency dependence of P3)—is related to freezing/melting of water in the core of the pores [31]. Sometimes this process, P3, is called the capillary transition. The process P2, was shown [31] to be independent on pore size, but strongly dependent on measurement frequency. This study related it to the dynamics of molecules which are attached to the pore walls. The third process P1 depends on the measurement frequency and pore size d [31,32]. In our previous works, preliminarily identification of this process with glassy freezing of water molecules at T**_g_**(d) within the pores was made, but it was not clear, where this water fraction was located and whether the core of ice influenced the freezing process, etc. This study shows new results of dynamic mechanical analysis (DMA) and thermomechanical (TMA) measurements of Vycor and Gelsil with pore diameters d=10 nm (V10), 5 nm (G5) and 2.5 nm (G2), partially filled with water. Combining these results with fully filled [31] and empty samples and with adiabatic calorimetric data [33], a rather coherent picture on the origin of the glassy behaviour of water in confined space has been obtained. In particular the present results suggest, that in confined water, the glassy freezing of water molecules occurs in a small spatial region of one or two layers which are located near the pore walls.

## 2. Results

### 2.1. Experimental

For DMA or TMA measurements, macroscopic samples of a few mm^3^ are needed. Therefore, this study cannot use e.g., porous silica powders, like MCM–41, etc., which usually are preferred because of their regular pore shapes and well-defined pore sizes. A good compromise for our purpose is to utilize mesoporous silica monoliths of Vycor and Gelsil which are described in more detail in Section 4.1. Vycor and Gelsil have different pore textures. In Vycor, the pores are of cylindrical shape, which are randomly distributed in length and orientation. The mean pore diameter d over pore length l was found [34] to be approximately d/l ≈ 0.23. These TEM investigations also yielded that all pores are open, but not fully interconnected. The production of Gelsil glasses involves a sol-gel process resulting in a different pore structure. The pores appear as voids between an assembly of stochastically arranged monodisperse silica spheres [35] which are touching and also penetrating each other. The voids between these spheres constitute a random network of inter-connected corridors and pockets, and show a larger pore size distribution as compared to Vycor. Furthermore, bottle-neck shaped and closed pores do arise.

To characterize our porous samples, mainly N_2_ adsorption-desorption measurements (BET/BJT-analysis [36,37,38]) were used. For Vycor (V10) and Gelsil (V5), this study also applied [39] small angle x-ray scattering (SAXS). From the analysis of the scattering data in the Porod regime, an average pore size of 11.1 nm for V10 and 5.2 nm for G5 was obtained, which is in reasonable agreement with the data from BET analysis. Table 1 summarizes the main specifications of these silica based mesoporous host materials as obtained [34,35,39] from N_2_ adsorption measurements.

A rough estimation—based on pore volumes and average size of water molecules—yields numbers of approximately 300, 2400 and 100.000 water molecules in 2.5 nm, 5 nm and 10 nm pores, respectively.

For thermal expansion measurements, a TMA 4000 (Perkin Elmer) as well as a Diamond DMA (Perkin Elmer) was used.

To study the slow dynamics of confined supercooled water, dynamic mechanical analysis (DMA) measurements were performed using two devices (DMA 8000 and Diamond DMA, Perkin Elmer).

With DMA, the complex Young´s modulus Y* = Y’ + iY’’ of a material as a function of temperature and frequency is essentially measured, as explained in detail in Section 4.2.

### 2.2. Experimental Results

**Figure 1:**
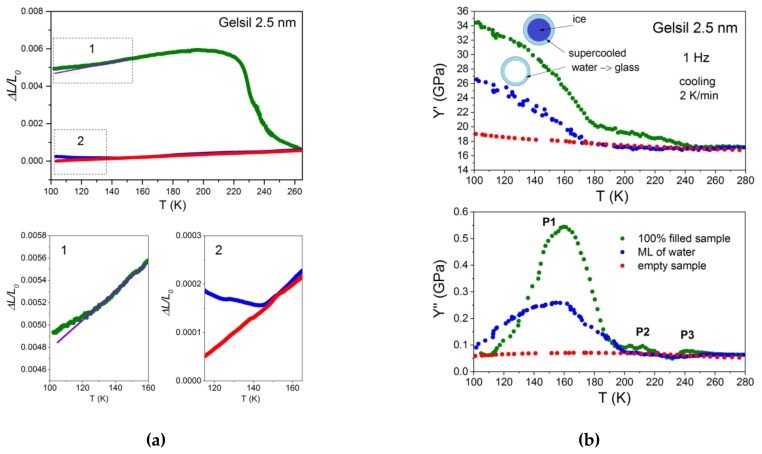
(**a**) Temperature dependencies of thermal expansion. Green (fully filled sample), blue (partly filled), red (empty sample). (**b**) real (Y′) and imaginary (Y′′) parts of the complex Young´s modulus of water in Gelsil 2.5 nm, measured at heating (heating rate 2 K/min) after the sample was slowly (2 K/min) cooled to 80 K. The results of samples fully filled with water (green), partly filled (blue) and empty samples (red) are compared.

In a foregoing study [32], the authors quenched the samples by dropping them directly into liquid nitrogen and measured the dynamic elastic response during heating. In this study, the authors used the moderate cooling/heating rates (2 K/min) and compared the results of the samples which were completely filled with water [31] to those where the pores were only partly filled and to samples with empty pores. Figure 1 shows the thermal expansion data as well as the real Y′ and imaginary Y′′ parts of the complex Young´s modulus of water in Gelsil 2.5 nm (G2) as a function of temperature for three different water fillings (100% filled sample, a few layers of water = ML, empty sample = ES). 

The procedure to obtain 100% filled samples, partly filled samples (ML = multilayers) as well as empty samples is described in Section 4.1.

In Figure 1, the following behaviour can be observed: Empty samples (red points) show no anomalies at the melting point P3 in thermal expansion ΔL/L_0_ as well as in Y′ and Y′′, as expected for the nanoporous silica matrix.

The completely filled samples (green points) exhibit, with decreasing temperature, a rather steep increase in thermal expansion and a moderate increase in Y′ due to the formation of ice (capillary transition), in the core [40,41] of the pores, and a very pronounced hardening in Y’ starting at approximately 180K, whose origin is discussed later. The onset of an ice formation is accompanied by a steep increase in ΔL/L_0_ and a peak (P3) in Y′′, which does not shift with frequency [32] as expected for a first order (freezing/melting-) phase transition.

By way of contrast, the peak P1 (adsorbate transition) in Y’’ shows a pronounced frequency dependence. It shifts to a lower temperature with decreasing frequency, as expected for a relaxational process and the temperature and frequency dependencies of Y′ and Y′′ can be well fitted [31] with a Cole-Cole function

(1) $$Y^{*}\left(\omega \right)={\;Y}_{\infty }-\frac{\Delta Y}{1+{\left(i\omega \tau \right)}^{\alpha }}$$

with α ≈0.28 and an Arrhenius dependence of the mean relaxation time

(2) $$\tau =\tau_{0}\exp\left(E_{a}/k_{B}T\right)$$

with an average activation energy of E_a_ ≈ 0.47 eV and τ_0_ ≈1.6 10^−15^ s for G2. In [31], the authors identified the process associated with P1 as the glass transition of a few layers of supercooled water close to the pore walls (see inset in Figure 1), which occurs in bulk water at the putative T_g_ ≈ 136 K. It is important to note that the thermal expansion of full samples shows (frame 1 of Figure 1a) at low temperatures only a very small anomaly starting at approximately140 K, whereas Y’ exhibits a large increase. This also reflects the dynamic nature of the process corresponding to P1. Indeed, inserting the values for the activation energy E_a_ and τ_0_ into Equation (2), it is found that τ(140 K)≈ 130 s. This also indicates that the system falls out of equilibrium around this temperature, as it is expected for glass.

It should be noted, that initially P1 was denoted [41] as the adsorbate transition of water in nanopores.

Figure 1 shows, that for partially filled samples (blue points) the peak P1 in Y’’ as well as the kink in Y’ occur practically at the same temperature as for fully filled samples (green points). As in the thermal expansion data, there is also no anomaly in Y′ and Y′′ at approximately 250 K (P3), proving that there is no formation of core ice in the pores. Similar to the full samples, the thermal expansion at low temperature shows a small anomaly starting at approximately 140 K (frame 2 of Figure 1a).

**Figure 2:**
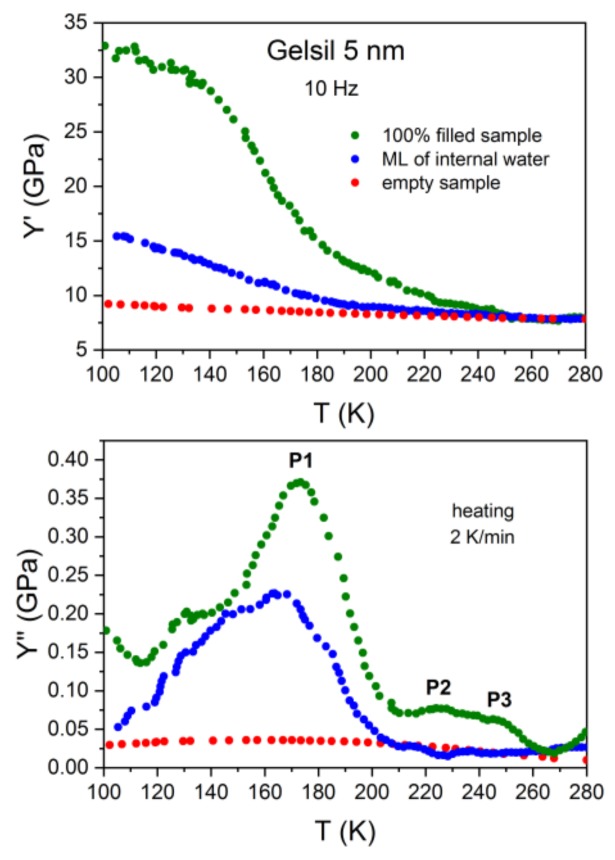
Temperature dependencies of real (Y’, top panel) and imaginary (Y’’, lower panel) parts of the complex Young´s modulus of water in Gelsil 5 nm, measured at heating (heating rate 2 K/min) after the sample was slowly (2 K/min) cooled to 80 K. The results of the samples fully filled with water (green), partly filled (blue) and empty samples (red) are compared.

**Figure 3:**
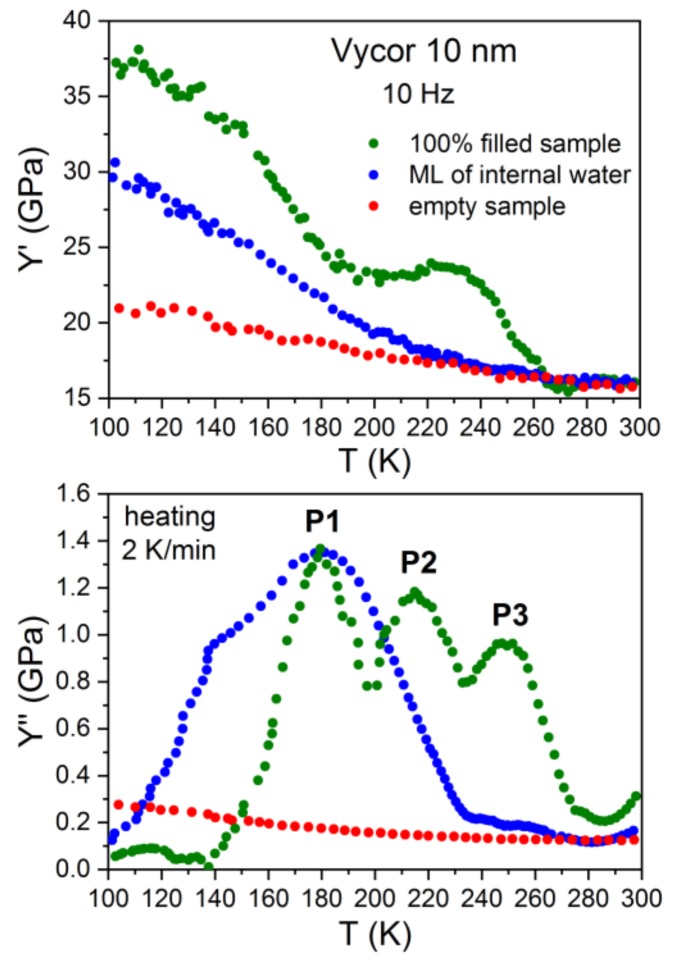
Temperature dependencies of real (Y’, top panel) and imaginary (Y’’, lower panel) parts of the complex Young´s modulus of water in Gelsil 10 nm, measured at heating (heating rate 2 K/min) after the sample was slowly (2 K/min) cooled to 80 K. The results of samples fully filled with water (green), partly filled (blue) and empty samples (red) are compared.

The same behaviour as demonstrated here for the case of water in Gelsil 2.5 nm has been found for G5 (Figure 2) and V10 (Figure 3) fully and partially filled with water. However, the anomalies concerning freezing/melting (P3) and the relaxation process P1 occur at higher temperatures [32], i.e., show a pronounced pore size dependence.

More than 40 years ago, mechanical relaxation measurements [41,42] of water in Vycor were performed at a single frequency of approximately 400 Hz. Taking into account the different frequencies, our results are in excellent agreement with these data. That is, calculating the position of P1 at f = 400 Hz (using our values obtained for the relaxation time) yields a value of T_P1_ = 188 K, which corresponds exactly to the temperature of the tanδ- peak of Ref.41. The authors give the following explanation for their measurements, which should also apply to our data: Based on earlier thermal expansion [43] and NMR [44] measurements of water in silica nanopores, the authors adopted a kind of "pea-in-pod" model, where just below the (pore size dependent) freezing temperature, T_f_ the system develops by forming ice in the centre of the pores surrounded by an unfrozen film of liquid water. Since in this temperature range the film of water is much less viscous than ice, it separates (mechanically) the ice from the silicon matrix, thereby diminishing the hardening effect of ice formation on the Young´s modulus. With further cooling, the viscosity of the adsorbed film of supercooled water increases gradually and finally cements the silica matrix and the ice together, resulting in the large increase in Young´s modulus (Figure 1, Figure 2 and Figure 3). However, the core-shell model of coexistence of water-layers and ice in pores larger than approximately 2 nm is well established [25,26,45], and the abovementioned explanation of the behaviour of Y′ seems to be out of the question.

**Figure 4:**
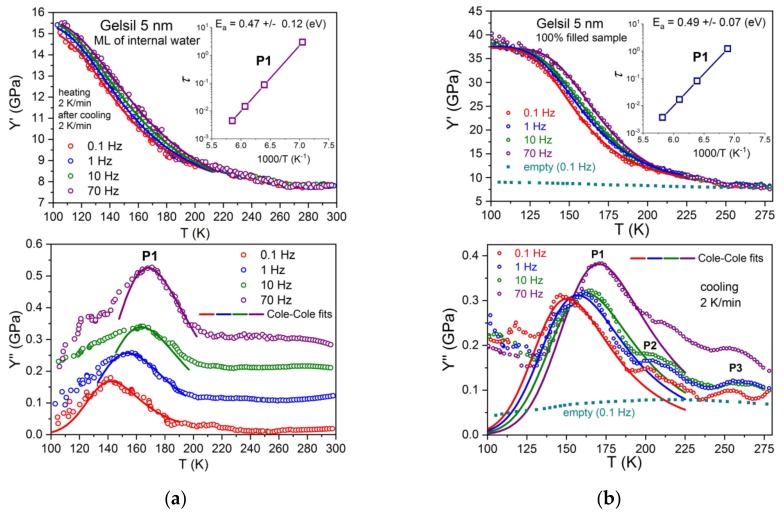
Temperature dependencies of Y′ and Y′′ at various frequencies: (**a**) partly filled samples (ML) of Gelsil 5 nm. (**b**) fully filled samples. The insets show Arrhenius plots corresponding to P1, yielding identical (within error bars) activation energies for samples partially and fully filled with water. Note, that the freezing/melting peak P3 does not shift with frequency. Y’’ data are shifted upwards to avoid overlapping of the data. Full lines are Cole-Cole fits using Equations (1) and (2).

To test for a possible influence of the core of ice on the thermal and dynamic behaviour of supercooled water in nanopores, we have performed detailed measurements of the dynamic elastic response of partially filled samples were performed and the results with those [31] of fully filled samples were compared. Figure 4 shows Y′ and Y′′ for partly filled samples (G5 is shown here as an example) and fully filled samples as a function of temperature and frequency.

Similar results have also been obtained for partly filled samples, G2 and V10. The typical effects of ice formation, i.e., the increase of modulus Y′ and the corresponding frequency independent peak P3 in Y′′ are absent in partly filled samples. Further, in the thermal expansion measurements, no sign of ice formation is detected. Therefore, the observed increase in modulus Y′ and the corresponding peak P1 in Y′′ can be safely associated with the processes occurring in the thin film (about two layers of water molecules) of supercooled adsorbed water in the pores. From the shift of P1 with the frequency and the Cole-Cole fits with an Arrhenius dependence of relaxation time, the activation energies (inset of Figure 4a) can be obtained which are in excellent agreement with the ones determined from fully filled samples (inset, Figure 4b). As already mentioned above, the frequency dependencies [31,32] of P1 have been measured for different pore sizes, yielding pore size dependent mean activation energies, i.e., E_a_ = 0.47 eV (G2), 0.49 eV (G5) and 0.52 eV (V10). Calculating the corresponding glass transition temperatures, by extrapolating the relaxation times τ = τ_0_exp (E_a_/k_B_T) to 100 s (to connect with calorimetric data [13,33]), a distinct pore size dependence of T_g_(d) was observed, which at d ->∞ approaches the traditional T_g_ ≈136 K.

**Figure 5:**
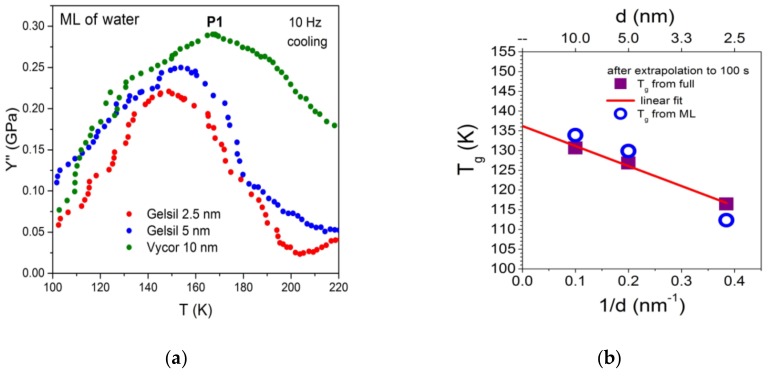
(**a**) Pore size dependence of the peak P1 for partly filled samples. The curves have been shifted for clarity. (**b**) Corresponding T_g_(d) values calculated from τ(T_g_) = 100s.

Interestingly enough, the peak maxima P1 in Y′′ and the activation energies E_a_ of partly filled samples show (Figure 5) a very similar pore size dependence as obtained for fully filled samples [32]. Thus, the same pore size dependence was obtained of the putative glass transition temperature T_g_(d) of supercooled water as for the fully filled samples, which extrapolates to 136 K for d ->∞. This finding has several implications. First it shows, that the liquid–to–glass transition in fully filled samples most probably occurs in the few layers of water molecules situated between the ice core and the pore walls. As a result, the observed distinct pore size dependence [31] of T_g_ is then related to the curvature of the pores rather than the pore diameter.

## 3. Discussion

In the following section, several scenarios are discussed for a description of the full set [31,32] of our data and this study attempts to ascertain which one describes them best in the light of existing models of confined water: Our data suggest that in partly filled and fully filled samples, a few (approximately two) layers of liquid water exist which are located near the pore walls and remain liquid until the lowest temperature, before glass freezing. This is mainly concluded from the fact that the relaxation behaviour of P1 for fully filled and partly filled samples yields identical activation energies. It is also in agreement with NMR measurements [46] of water in Vycor 10 nm and Gelsil 5 nm and 2.5 nm, which revealed that in fully filled samples, water crystallizes only in part in the core of the pores and an interface layer with a thickness of h ≈ 0.5 ± 0.1 nm remains liquid, independent on the geometry of the porous matrix and the pore size distribution. The viscosity of these approximately two layers of supercooled water increases with decreasing temperature, thereby producing the relaxation peak P1 in Y′′ and the corresponding increase in the Young´s modulus Y′ (Figure 1, Figure 2, Figure 3 and Figure 4).

There is general consensus [47] that the viscosity of supercooled water increases with decreasing temperature. However, the opinions are diverging when it comes to the interpretation of the increase in viscosity of the supercooled layers. One study associates it with the liquid-to-glass transition of supercooled water in confinement. Another study [10,11] questions such an interpretation. They relate the increase of viscosity to a freezing of proton reorientations at the lattice defects [18,42], presumably of the Bjerrum type. The liquid-to-glass transition—connected with the freezing of long-range diffusion of water molecules—would then occur at a higher temperature, which some authors [18] identifying preliminarily with the strong-to-fragile transition of water occurring at approximately 225 K.

Indeed, early mechanical relaxation measurements [3] of ice revealed that proton reorientation motion is a thermally activated process that is well described by an Arrhenius law with activation energy of E_a_ ≈ 0.5 eV, which is practically identical with the activation energy of the process usually associated with the glass-to-liquid transition of water. Thus, only from the dynamical point of view alone, it is virtually impossible to discriminate between a reorientation unfreezing and a glass-to-liquid scenario. However, taking into account that it is impossible to nucleate ice in a system smaller than approximately1 nm [46], and knowing that in confinement the liquid film consists of approximately two layers (0.7 nm) of supercooled water, it seems very unlikely that the observed relaxation peak P1 in partly filled samples (and due to the above mentioned similarities also in fully filled samples) can be attributed to just water reorientations in a kind of pseudo-crystalline environment.

Moreover, it is very difficult to accept that the unfreezing of proton reorientation could produce such a large softening in Young´s modulus as has been observed (Figure 1, Figure 2, Figure 3 and Figure 4) in fully and partly filled samples, while there is only a very tiny change in thermal expansion in this temperature range. Previously, the authors have found [48] that a plastic crystal, as e.g. C_60,_ yields some change in Y’(T) = Y^∞^− (Y^∞^ − Y^0^)F(ωτ), when going from the limit ωτ(T) < 1 to ωτ(T) > 1 due to freezing of some orientational degrees of freedom. However, the strong decrease (observed here for supercooled water in pores) in Y’ to a value which is close to the background value of Y’ for empty samples would imply that the orientationally disordered network of water layers—which is frozen with respect to the position of molecules—would be extremely soft. More work is needed to test such a possible behaviour experimentally or by computer simulations.

If, on the other hand, it is assumed that the phenomenon of P1 (i.e., the adsorbate transition) is not due to re-orientational unfreezing of water molecules, the following scenario can be proposed, which in the authors’ opinion, rather naturally describes our data: It is well known that water cannot crystallize [46] if the dimension of the confinement is less than a critical size d* ≈ 1 nm. In the present samples, either fully filled or partially filled with water, there are approximately two layers [45] of water (approximately h ≈ 0.7 nm thick, since a water molecule has an effective diameter [49] of approximately 0.38 nm) that cannot crystallize, since h < d*. Below the low temperature stability limit T_s_ ≈ 235 K (the homogeneous nucleation temperature), water inevitably should transform into ice. However, since in the present situation the thin film of water is confined between the core of ice and the pore walls (for fully filled samples) or just two layers thick (for partially filled samples), it cannot form a stable ice phase at any temperature. As a result, the system can occupy two metastable states, i.e., supercooled water (higher in energy space) and metastable ice-like states (lower energy states), separated by an energy barrier E_a_. At high temperatures, the relaxation time τ=τ_0_exp(E_a_/k_B_T) is short allowing the system to take equilibrium occupation probabilities of the two states. At low temperatures, τ is very large, resulting in a frozen occupation of the liquid state. The glass transition occurs at a temperature T_g_, when τ(T_g_) is approximately 100 s, i.e., the system falls out of equilibrium. Using a similar two-states model, the orientational glass transition of C_60_ was successfully described [48,50].

**Figure 6:**
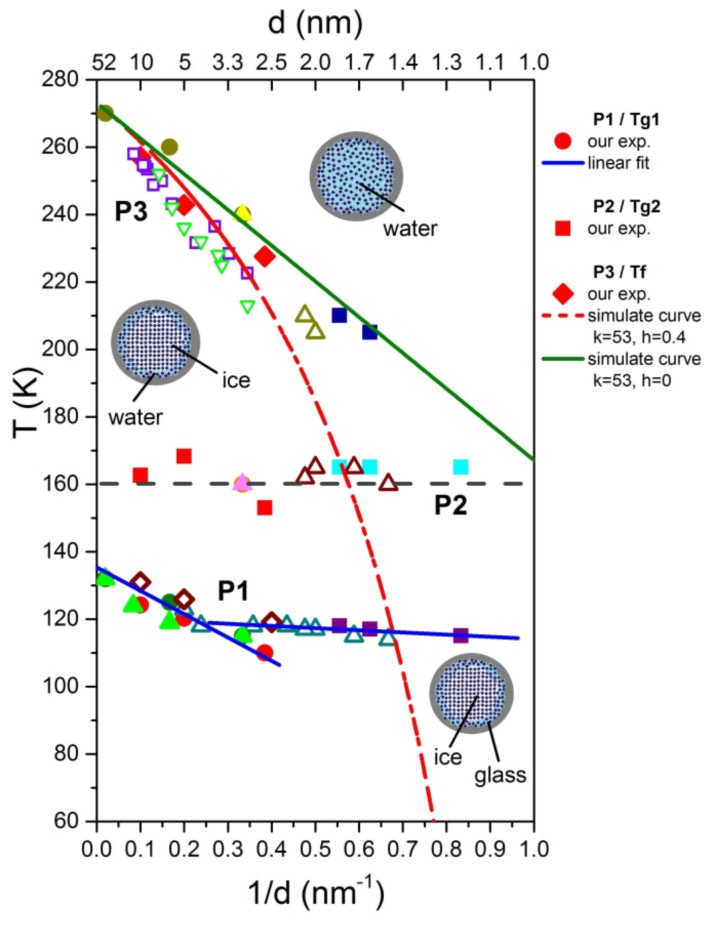
Pore size dependencies of the processes P1, P2 and P3 from DMA measurements (red symbols) and adiabatic calorimetric data [13,33]. Reproduced from the data of Figure 6 from Ref. [31], where all symbols are explained in detail. P3 = melting line, P1 = glass transition, P2 = due to re-orientational motion of water molecules attached to pore surface by hydrogen bonding [31]. The red dot-dashed line is calculated using [27] T_m_(d)=T_m_^bulk^ – K_GT_/(d/2-h), with K_GT_= 53 Knm and h=0.4 nm. The green line is calculated with h=0.

Recently, G. Floudas studied the kinetics of ice nucleation of water confined in nanoporous alumina. They found [51] that, prior to crystallization, undercooled water molecules relax with an activation energy of E_a_ ≈ 0.52 eV. This value, which is very similar to the one found for our process P1, corresponds to the formation of a few hydrogen bonds, and can therefore be seen as a precursor to ice nucleation. However, due to the confined space, these nuclei cannot grow to the critical value of approximately 1 nm. Thus, in this picture, the relaxation peak P1 originates from a freezing process, which occurs as a result of geometrical frustration, i.e., the system cannot reach the ground state (ice) due to the confined space and gets caught at T_g_ in the higher energy state. Indeed, looking at Figure 6, which is reproduced from Figure 6 of [31], it can be observed that the freezing/melting line of water crosses the glass transition temperature at approximately d ≈ 1.4 nm. It shows that with decreasing pore size, the freezing/melting temperature of water decreases according to the well-known modified Gibbs-Thomson equation, but at pore diameters smaller than approximately 1.4 nm, a glass transition takes place instead of ice formation.

Limmer and Chandler [52] have extended the phase diagram of supercooled water confined in hydrophilic nanopores to the regime of very small pores, showing that crystallization is suppressed for a pore radius R < l_s_ ≈ 0.9 nm due to the enhancement of fluctuations. For pore diameters < 2 nm, these authors predict (see Figure 2 of [52]) a transition from liquid to glass with a much weaker pore size dependence as for the liquid-ice transition taking place at d > 2 nm, which is in very good agreement with the experimental data (Figure 6).

## 4. Materials and Methods

### 4.1. Meso-Porous Materials

As already mentioned above in Section 2.1., relatively large samples are needed for measurements. For this reason, this study could not use the preferred ordered mesoporous silica materials e.g., MCM-41, SBA-15or other controlled porous glasses (CPGs) with pores of uniform size, which are available only in powder. Instead, monoliths of mesoporous silica, i.e., Vycor and Gelsil, were utilized.

The porous glass sold under the brand name Vycor 7930 by Corning Inc. (Corning, NY, USA), NY arises from a temperature induced phase separation within a Na_2_O-B_2_O_3_-SiO_2_ melt. After cooling the Ba_2_O_3_-rich phase is leached out with an acidic solution, which leaves a 96% pure SiO_2_ skeleton [34]. The pores are cylindrical and randomly distributed in length, density and angle. The leaching process ensures that all pores are open, but not fully interconnected as pockets show up in TEM pictures [34]. The pore sizes can be varied by intercepting the phase separation process.

Gelsil monoliths are produced in a sol-gel process by hydrolization of silica containing precursor liquids, followed by condensation and heat treatment. Various precursors with different stabilizers (i.e., organic molecules) are in use to create highly porous Aerogels, Xerogels like Gelsil or highly hierarchical organized porous silica [53]. Silica molecules condensate to spheres at stochastic sites within the hydrolized silica precursor. The subsequent gelation leads to a network-like arrangement of spheres. From the heat treatment, the gel turns either into a bulk-like powder or monoliths. Thus, the dried and consolidated end product can be approximated as an assembly of stochastically arranged and monodisperse pure silica spheres [35]. The spheres are touching and also penetrating each other. The voids between these spheres constitute a random network of inter-connected corridors and pockets and show a larger pore size distribution compared to Vycor 7930. Further, the bottle-neck shaped and closed pores do arise. The porosities range from 0.5 <φ< 0.9, and the densities are typically 1 <ρ< 1.5 g/cm^3^.

Until the late 1990s, a company called Gel Tech Inc. located in Alachua, Florida is an often-cited supplier. However, in 2001, the production of Gelsil ceased. After weeks of searching, this study discovered that there is only one company still producing Gelsil, which is 4F International Co. in Gainesville, FL, USA.

To prepare the samples for DMA or TMA measurements, Vycor and Gelsil with typical sizes of 3 × 2 × 2 mm^3^ were cut with a diamond saw and sanded to gain parallel surface plains. Then, the bars were cleaned by first dropping them into a 30% H_2_O_2_ solution at 90°C for 24 h, followed by drying at 120°C in a high vacuum chamber also for 24 h.

The complete filling of the samples was done by making use of the strong capillary forces of the narrow pores, by dropping the sample on one end (to avoid air-bubbles) into water until the opaque empty sample gets fully transparent. For partly filled samples (ML = multilayers), the following procedure was applied: First, the samples were fully filled. Then, they were heated up to 25°C (for Vycor 10 nm), 35°C for Gelsil (5 nm) and 38°C for Gelsil (2.5 nm) and kept at these temperatures for 10 min. The empty samples were produced by heating them up to 150°C for 10 min. The temperature dependent measurements of thermal expansion (Figure 1a) proved that the samples were partly filled (i.e., no thermal expansion anomaly due to the formation of ice in the pores) or completely free of water (no thermal expansion anomaly at all), depending on the foregoing procedure.

### 4.2. Dynamic Mechanical Analysis

In both devices (DMA 8000 and Diamond DMA, Perkin Elmer, Waltham, MA, USA), a dynamic force F_D_.sin(ωt) is applied in addition to a static force F_s_. The real Y’ and imaginary Y’’ parts of the complex Young´s modulus Y*=Y’+ iY’’ are determined from the measured sample strain ε=ΔL/L_0_ and phase shift δ between the externally applied dynamic force F_D_ and the sample strain using the relation:

(3) $$Y\prime \;=\;\frac{F_{D}}{A\varepsilon }cos\left(\delta \right)\;\mathrm{and}\;Y\prime \prime \;=\;\frac{F_{D}}{A\varepsilon }sin\left(\delta \right)$$

where A is the sample area, which in parallel plate geometry is in contact with the tip of the DMA apparatus.

The measurement frequency can be varied between 0.01 Hz and 100 Hz at temperatures between 80 K and 870 K. A force up to 10 N can be applied, with a resolution of 0.002 N. The resolution of the sample height is approximately3 nm and the phase shift δ can be measured with an accuracy of approximately 0.1°. The relative accuracy of the DMA method is approximately 0.2–1%, but the absolute accuracy of such a measurement is usually not better than approximately 20%. To obtain reasonable absolute values, the measured Y’-data was normalized at room temperature to the Young´s modulus data previously measured [38] by RUS (resonance ultrasonic spectroscopy). The Y’’ values were then obtained from the phase shift data δ using Y’’= Y’tanδ.

More details about the experimental method can be found in [54].

## 5. Conclusions

Based on the data of thermal expansion and complex mechanical relaxation measurements of different amounts of supercooled water in mesoporous silica (Vycor, Gelsil), this study claims that confined water exhibits glassy behaviour, which is thought to be due to the suppression of crystallization in small pores. It can be called a geometrically frustrated glass for the following reason. It is generally believed that in pores larger than approximately 2 nm in diameter, a core of ice is formed in the centre of the pores with decreasing temperature. This leaves in practically all pores, a thin film of supercooled liquid water with nearly the same thickness of approximately 0.7 nm (approximately 2 layers of water molecules). This size is smaller than the critical size for the formation of a stable ice nucleus. Indeed, Figure 6 could be interpreted in the following way: Ice is formed in the core of the pores, and the freezing temperature (P3) decreases with decreasing pore size. At a critical size of approximately —d ≈1.4 nm, the freezing/melting line crosses the line of P1, i.e., crystallization stops, when the freezing temperature T_f_(d) becomes of the order of the glass transition temperature T_g_. The fact that the samples with a few layers of water show nearly the same relaxation behaviour of P1 (Figure 1, Figure 2, Figure 3, Figure 4 and Figure 5), strongly indicates that the liquid-to-glass behaviour in small pores takes place in a few layers of water near the surface of the pores. It also suggests that the distinct pore size dependence of T_g_ between 2.5 nm and 50 nm for fully filled samples (Figure 6 and [31]), as well as for partially filled samples (Figure 5) is not a real pore size effect, but should be rather interpreted as an effect of curvature.

A two states model, similar [50] to that of C_60_, between the states of supercooled water (higher in energy) and crystal-like states (lower in energy but also metastable due to supressed crystallization) describes our data, and with it, probably also many other results on confined water quite well. It yields glass-like anomalies in specific heat at T_g_(d) if (dT/dt)τ(T_g_)/T_g_ ≈ 1 and dielectric and elastic relaxation peaks that shift with the frequency resulting in the mean activation energies of approximately 0.5 eV. It also describes, very naturally, the strong softening observed in Y’ (Figure 1, Figure 2, Figure 3 and Figure 4) when going from T < T_g_ (majority of molecules is frozen states) to T > T_g_ (an increased fraction of liquid-like states).

## Figures and Tables

**Table 1:** Characteristic data of mesoporous silica monoliths of Vycor and Gelsil.

Properties	V10	G5	G2
Pore size (nm)	10 ± 0.5	5 ± 0.8	2.5 ± 1
Porosity (%)	40	54	36
Surface/volume ratio	4.5	8.3	15
specific BET surface area (m^2^/g)	90	510	590
Pore volume(cm^3^/g)	0.4	0.54	0.38
